# Numerical Simulation and Accuracy Verification of Surface Morphology of Metal Materials Based on Fractal Theory

**DOI:** 10.3390/ma13184158

**Published:** 2020-09-18

**Authors:** Xiaokai Mu, Wei Sun, Chong Liu, Bo Yuan, Yunlong Wang, Qingchao Sun

**Affiliations:** School of Mechanical Engineering, Dalian University of Technology, Dalian 116024, China; sunwei@dlut.edu.cn (W.S.); chongl@dlut.edu.cn (C.L.); yuanbodut0411@mail.dlut.edu.cn (B.Y.); yunlong225@mail.dlut.edu.cn (Y.W.)

**Keywords:** metal materials surface, parameters characterization, fractal theory, numerical simulation, morphology substitution

## Abstract

This paper presents a numerical simulation method to determine the surface morphology characteristics of metallic materials. First, a surface profiler (NV5000 5022s) was used to measure the surface, and the morphology data thereof were characterized. Second, fractal theory was used to simulate the surface profile for different fractal dimensions *D* and scale coefficients *G*, and statistical analyses of different surface morphologies were carried out. Finally, the fractal dimension *D* of the simulated morphology and the actual morphology were compared. The analysis showed that the error of fractal dimension *D* between the two morphologies was less than 10%; meanwhile, the comparison values of the characterization parameters of the simulated morphology and the actual morphology were approximately equal, and the errors were below 6%. Therefore, the current method used to evaluate the surface morphologies of parts processed by the grinding/milling method can be replaced by the simulated method using the corresponding parameters. This method makes it possible to theorize about the surface morphologies of machined parts, and provides a theoretical basis and reference value for the surface morphology design of materials, with the potential to improve the assembly quality of products.

## 1. Introduction

In mechanical engineering, the surface profile height of machined parts, which is affected by the process of machining and wear, is nonstationary and random [1]. From a microscopic point of view, the morphology surface is composed of several asperities, showing complex morphological characteristics. Because mechanical products are composed of several different parts according to certain requirements, and the contact between the mating surfaces is actually made up of several asperities, the surface morphology characteristics of the parts have a direct impact on the assembly quality and the performance of the product. Therefore, accurate assessments of surface morphology are of great significance for quality control and performance predictions.

As we know, no machined surface is perfectly smooth; there is always a certain surface roughness. When a surface is enlarged to a certain scale, many uneven microconvex bodies can be seen. At the microscale, the contact interface between two rough surfaces actually involves contact between a series of microconvex bodies, which has contact heterogeneity [2], so the surface micromorphology of the part is closely related to the contact performance between the mechanical interfaces. Therefore, finding a numerical method to effectively simulate rough surface morphologies is necessary to accurately determine the correlation between the morphology characteristics and the contact performance of the interface.

In the era of rapid development of computer technology, numerical simulation technology has become an important means in tribology research [3]. Due to its strong randomness, complex formation processes and many influencing factors, the quantitative study of surface morphologies mainly depends on data analysis after measurement, i.e., postanalysis. In fact, the surface morphology always changes in the contact process. The evolution of the surface morphology is transient in time and discrete in space, which makes it difficult to analyze the impact of the joint surface on the contact performance. This requires that the relevant data be simulated and analyzed using numerical methods to obtain a large amount of effective data; then, the influence of the morphology characteristics on the contact performance can be predicted in advance, so as to realize a prior analysis. Therefore, the ability to accurately analyze the contact performance of joint surfaces and effectively improve assembly quality using a numerical method to yield accurate characterizations of surface morphologies is of great importance.

At present, surface characterizations of rough surface mainly use quantitative descriptions based on statistics. The surface characteristic parameters obtained by this method are obviously limited by the resolution and sampling length of the instrument, and do not have objective uniqueness, so it is difficult to fully reflect the random behavior and detailed characteristics of rough surfaces, giving rise to many inconveniences in practical application [4]. In the early days, research on surface structure was limited to surface roughness, which was used to study the relationship between the surface morphology and the contact performance of parts in order to provide accurate quantitative descriptions of surface structures [5]. Since the appearance of the first surface morphology measuring instrument in the 1920s, a great deal of quantitative research on the evaluation parameters of surface morphologies has been undertaken [6]. To date, no uniform standard for evaluation parameters has been adopted internationally, and the repetition rate is high, which leads to the so-called “parameter explosion” phenomenon described by Whitehouse [7]. The most common morphology characterizations are carried out on the two-dimensional contour lines of a normal section of surface, using the midline system and only a few evaluation parameters, such as the arithmetic mean deviation *R*a, the mean square deviation *Rq*, etc. [8]. Dong et al. [9] divides the basic parameters of surface morphology characterization into four groups, i.e., amplitude parameters, spatial parameters, mixing parameters and functional parameters. Traditional morphology characterization is based on the median filtering method to separate the high- and low-frequency surface information. The sampling length is the equal interval length of the original contour, e.g., the least squares median and the arithmetic mean median [10,11], which only considers the influence of interval, without considering the local characteristics of surface morphology. In addition, the polynomial fitting and spline fitting methods are similar to the median filtering method [12,13,14]. If the sampling length is too short, roughness information will be lost; otherwise, roughness evaluations will be affected by waviness. Due to the shortcomings of traditional characterization methods, superior surface morphology evaluation methods such as the motif method, power spectral density, wavelet transform and fractal theory have appeared in recent years, which are useful supplements to traditional methods [15,16].

In summary, the characterization of a rough surface is mainly based on the quantitative description method using statistics. Because the number of statistical parameters is limited, there will be some deviation in the characterization of some local details and the random characteristics of the surface morphology. Therefore, using traditional parameters to describe the profile does not accurately express the real characteristics of the surface.

In the machining process of parts, which is affected by the parameters of the cutting tools, machine tools, cutting parameters and other factors, the machined surface is not an absolutely ideal smooth plane, but is composed of a series of random micromorphology features which show strong continuity, nondifferentiability and self-affinity in mathematics [17,18,19]. Therefore, the machined surface can be regarded as a microgeometry combination that satisfies the characteristics of a fractal. At present, many researchers have carried out related research to the analysis and numerical simulation of surface micromorphology characteristics based on fractal geometry theory. Sayles and Thomas [20] pointed out that the height distribution of rough surface morphology has an unstable random characteristic and a certain relationship with the length of the sampled morphology. Majumdar and Bhushan [21] pointed out that surface roughness has fractal characteristics from the nanometer to the centimeter scale, that is, self-similarity and self-affined, and that the fractal characteristics of rough surface profiles have no relationship with the profile scale. Hasegawa et al. [22] calculated and counted the surface morphology parameters of a grinding surface, and pointed out that the surface obtained by grinding has fractal characteristics within a certain range. Liu et al. [23] pointed out that surface micromorphologies have nonstationary and multiscale characteristics, and went on to say that the micromorphology of parts can be characterized by the fractal dimension of different parameters. Persson [24] analyzed the microcharacteristics of surface profiles, and realized the simulation of different contour curves with different fractal dimensions. On this basis, the validity of the profile simulation method was verified by comparing the key parameters of the simulated profile and the measured profile. Liu et al. [25] analyzed the microscopic characteristics of surface profiles and then used mathematical methods to build related surface models with different fractal dimensions. Ruello [26] and Jackson [27] proposed an effective method to construct a microsurface model using the fractal method which can characterize profiles at different scales. Kulesza and Bramowicz [28] proposed a fractal surface mathematical model which can quantitatively describe the self-similar microstructures of surfaces by the fractal geometry and angle measurement analysis methods. Almqvist et al. [29] used different methods to simulate surfaces, and verified the accuracy of their simulations by comparing the simulated surface with the actual surface. Feng et al. [30] realized the effective reconstruction of a surface morphology at different scales through fractal theory, which provided an effective model for studying the contact performance of the interface.

In summary, fractal theory in the characterization process of surface morphologies is gradually gaining popularity, as it can characterize the complexities of surface morphologies, has strong descriptive stability and is not limited by sampling length. However, little research has been undertaken on the characterization of rough surface morphologies based on measured data. Therefore, researching the fractal simulation method of rough surfaces based on measured data is an effective way to analyze the influence of surface morphology on the interface contact performance and improve product performance.

**Figure 1:**
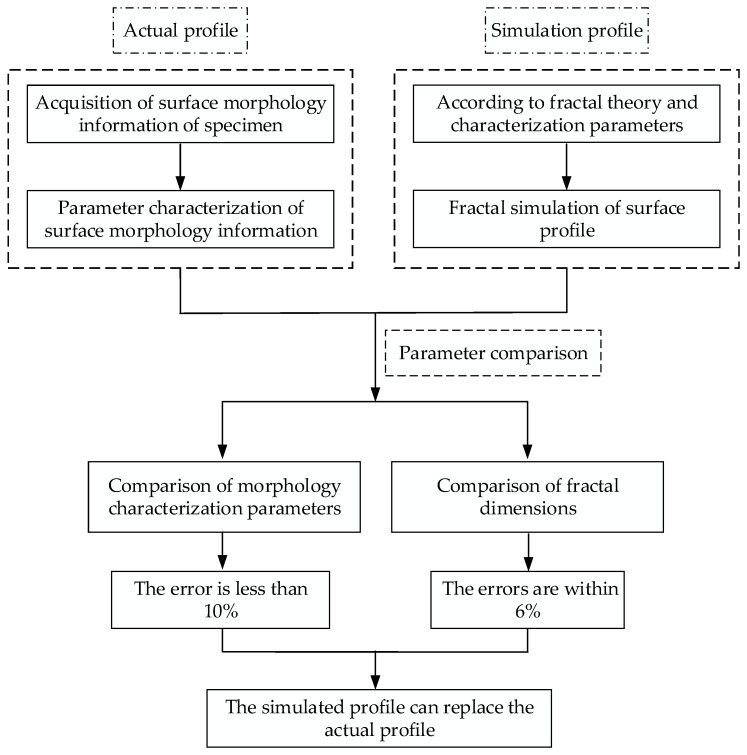
The article block diagram.

In order to realize an effective representation of the actual part surface and solve the problem that the surface parameters in traditional statistics cannot completely and accurately reflect the real part surface characteristics and multiparameter limitations due to the influence of measurement conditions (such as the resolution and sampling length of the profilometer), this paper uses fractal theory to simulate the surface profiles of different fractal dimensions *D* and scale coefficients *G* based on the measured grinding and milling surface morphology data, and then characterizes the parameters and presents a statistical analysis. On this basis, the consistency between the theoretical and the actual morphology was verified by comparing the morphological characterization parameters and fractal dimension *D*; in this way, consistency between the theoretical and the real morphologies is finally achieved. A block diagram of the structure of the article is shown in Figure 1. The proposed method makes it possible to theorize about the surface morphology of machined parts, and lays a foundation for the analysis of the relationship between the morphology characteristics and the interface contact performance, thereby potentially improving surface morphology designs and product performance.

## 2. Acquisition and Parameters Characterization of Surface Morphology

Due to hardware limitations, the characterization of real contact surface morphologies mainly uses traditional mathematical methods, and cannot really reflect the micromorphology characteristics of rough surfaces. In this section, samples with different processing methods are taken as the objects, and the surface morphology data are obtained by testing and characterized by parameters, which provides the data basis for numerical simulation and effective verification of surface morphologies.

### 2.1. The Acquisition of Surface Morphology

#### 2.1.1. Specimen and Test Equipment

**Figure 2:**
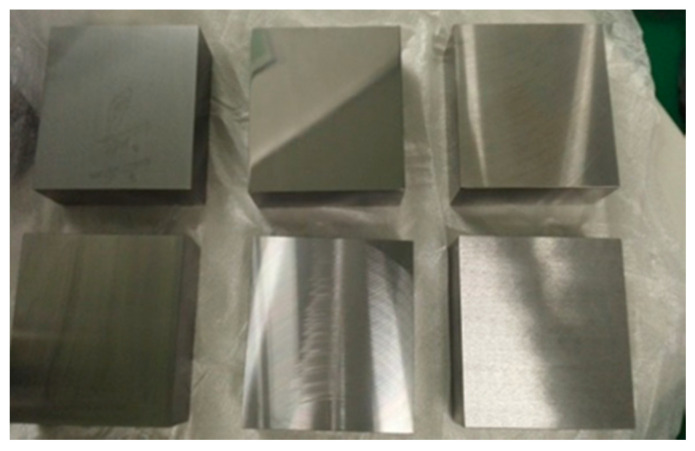
Specimens.

**Figure 3:**
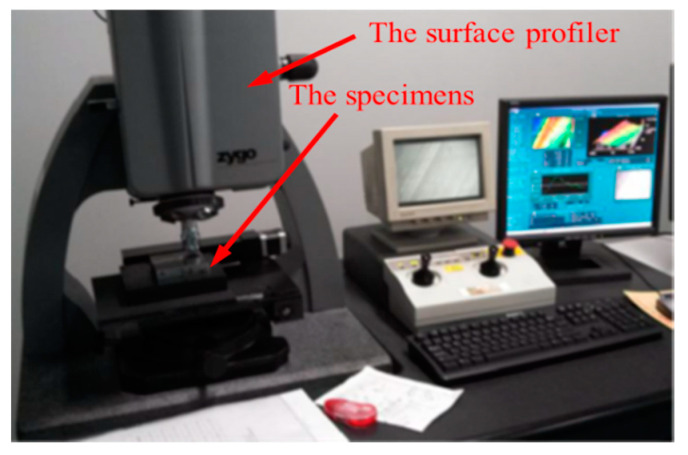
The test instrument.

The specimens were processed in a suitable factory. Their material was C45 steel, with dimensions of 80 mm × 80 mm × 30 mm, obtained by the milling and grinding methods. Surface morphology data were obtained by using a surface profiler (NEW view 5022) manufactured by ZYGO (Middlefield, CT, USA). The instrument uses the principle of white light interference to detect the microgeometric characteristics of the surface; its vertical resolution is 0.1 nm and lateral resolution is 110 nm. The specific specimen and the test instrument are shown in Figure 2 and Figure 3, respectively.

#### 2.1.2. Expression of Test Data

**Figure 4:**
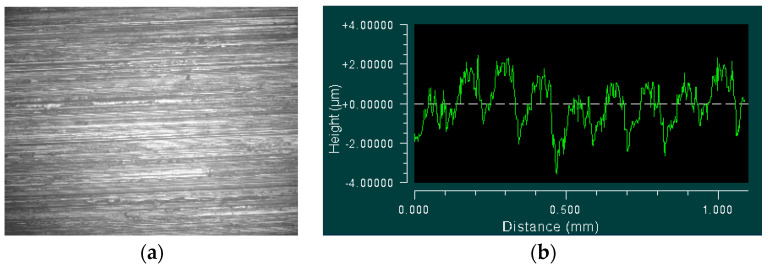
Measured surface and profile data (**a**) Specimen surface and (**b**) Measured profile curve.

In order to reflect the true surface morphology, this paper measured the upper and lower surfaces of each specimen, and extracted two-dimensional surface profile data from the measured morphologies, which provided the basis for comparisons with the simulated profile data. The specimen surface and measured profile data are shown in Figure 4.

**Figure 5:**
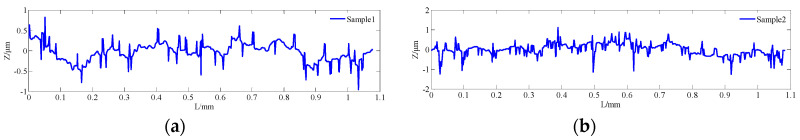

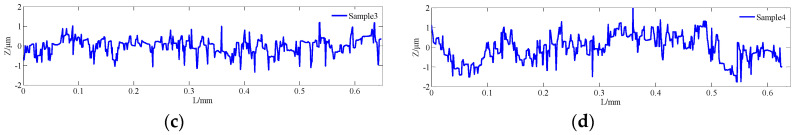
Actual surface profile curves of different processing methods (**a**) The upper surface of specimen 1 machined by grinding; (**b**) The lower surface of specimen 2 machined by grinding; (**c**) The upper surface of specimen 3 machined by milling and (**d**) The lower surface of specimen 4 machined by milling. (**a**) the upper surface of specimen 1 machined by grinding.

The surface profiler was used to test the specimens with different processing methods. The obtained surface profile curve is shown in Figure 5.

### 2.2. Parameter Characterization of Test Data

The surface morphology of parts is critical to the matching quality and the contact performance of the product, affecting the friction and wear properties between the mating surfaces to a certain extent [31,32]. Parametric characterization of the surface geometry is an effective way to accurately predict the relationship between the surface morphology and the contact performance of the joint surface and to realize numerical simulations of the surface morphology.

#### 2.2.1. Characterization Parameters of Morphology Data

**Figure 6:**
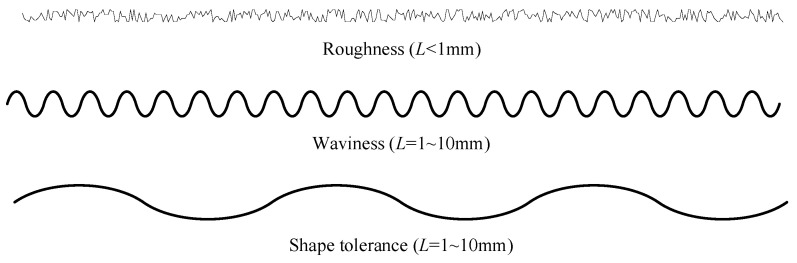
Decomposition diagrams of the surface morphology.

As shown in Figure 6, the surface geometric characteristics of parts have certain randomness. Therefore, random process parameters (the mean square value, the mean square deviation, the autocorrelation function and the probability density) are often used as the main characterization parameters of surface profiles.

*R_a_* is the arithmetic mean deviation of profile, which is the arithmetic average value of the height of each point on the profile within the measuring length range

(1) $$R_{a}=\frac{1}{L}\int_{0}^{L}\left|z(x)-m\right|dx=\frac{1}{n}\sum_{i=1}^{n}\left|z_{i}-m\right|$$

where *m* is the average value of the profile data points.

The mean variance *σ* and mean square deviation *R_q_* of profile peak height can be obtained as:

(2) $$\sigma^{2}=\frac{1}{L}\int_{0}^{L}{\left[z(x)-m\right]}^{2}dx$$

(3) $$R_{q}^{2}=\frac{1}{L}\int_{0}^{L}{\left[z(x)\right]}^{2}dx$$

so

(4) $$\sigma =\sqrt{\frac{1}{L}\int_{0}^{L}{\left[z(x)-m\right]}^{2}dx}=\sqrt{\frac{1}{L}\sum_{i=1}^{n}\int_{0}^{L}{\left(z_{i}-m\right)}^{2}}$$

At present, *R_a_* and *σ* are the main description parameters of surface roughness characteristics. However, due to periodic and random changes in the surface morphology of the parts, it is more scientific to describe the surface geometric characteristics by using shape statistical parameters than a single shape parameter, as they can effectively reflect the height, wavelength and curvature of each point on the profile curve.

According to [4], the surface profile data of machined parts approximately follow the Gauss distribution law, and the probability density function is:

(5) $$\psi (z)=\frac{1}{\sqrt{2\pi }\sigma }\exp\left(-\frac{z^{2}}{2\sigma^{2}}\right)$$

where *Ψ*(*z*) represents the probability of different profile heights. Theoretically, the range of the Gauss distribution curve is from −∞~+∞. In fact, 99.9% of the distribution is included between −3*σ* and +3*σ*. Therefore, the error caused by taking ±3*σ* as the limit of Gauss distribution can be ignored.

In addition, the autocorrelation function *R*(*τ*) can be used in the analysis of surface morphology parameters; it can express the internal relationship of the adjacent profile peaks and the change trend of the profile curve.

For a profile curve, the autocorrelation function is the mathematical expectation (average) value of the product of the profile height *z*(*x*) at each point and *z*(*x* + *τ*) with the distance *τ*,

(6) V(τ)=E[z(x)z(x+τ)]

where *E* is the mathematical expectation.

If the measuring point in the measuring length *L* is n and the coordinates of each measuring point are *x_i_*, then *V*(*τ*) is

(7) $$V(\tau )=\frac{1}{n-1}\sum_{i=1}^{n-1}z(x_{i})z(x_{i}+\tau )$$

For the profile curve of continuous function, the above formula can be written as follows:

(8) $$V(\tau )=\underset{L\to \infty }{\lim}\frac{1}{L}\int_{-L/2}^{+L/2}z(x)z(x+\tau )dx$$

The autocorrelation function is a comprehensive index reflecting the height and effective wavelength of the surface profile, which is very important for studying the change of surface morphology.

#### 2.2.2. Characterization of Morphology Data

According to the above contents, the measured surface profile curve can be characterized by the relevant parameters, as shown in Table 1.

**Figure 7:**
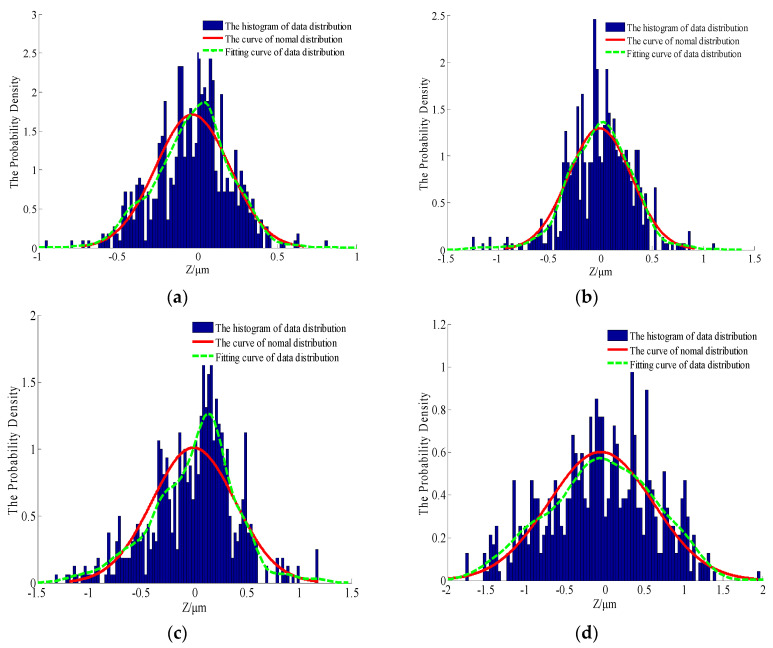
The probability density graphs for different surface morphologies (**a**) The upper surface of the specimen 1; (**b**) The lower surface of the specimen 2; (**c**) The upper surface of the specimen 3 and (**d**) The lower surface of the specimen 4.

**Figure 8:**
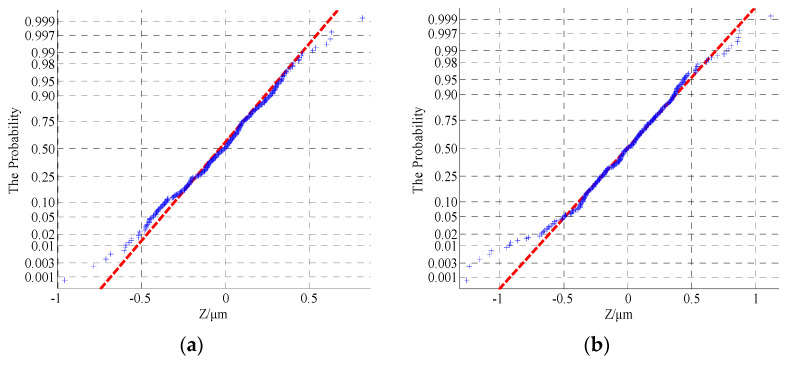

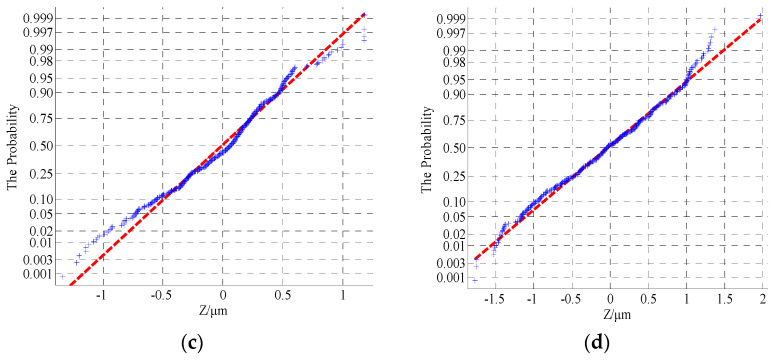
QQ test figure for different surface morphology (**a**) The upper surface of the specimen 1; (**b**) The lower surface of the specimen 2; (**c**) The upper surface of the specimen 3 and (**d**) The lower surface of the specimen 4.

**Figure 9:**
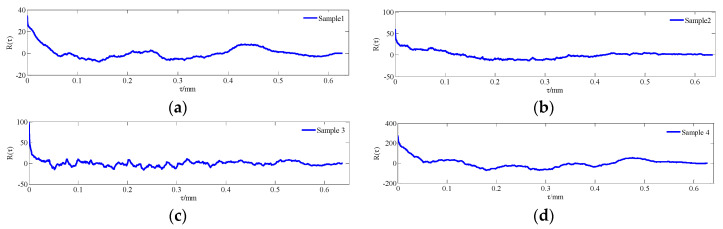
Autocorrelation graphs for different surface morphologies (**a**) The upper surface of the specimen 1; (**b**) The lower surface of the specimen 2; (**c**) The upper surface of the specimen 3 and (**d**) The lower surface of the specimen 4.

Due to the limitation of one-dimensional morphology parameters in the expression of real surface morphologies, probability density statistics and autocorrelation analyses of measured data can more accurately reflect the distribution rule and the relevant characteristics of surface morphologies; this principle will lay a foundation for the effective verification of simulated morphologies. The relevant statistics of the measured data are shown in Figure 7, Figure 8 and Figure 9.

As shown in Figure 7 and Figure 8, the surface profile data of the actual machined parts are basically conformed to show normal distribution. At the same time, it can be seen from Figure 9 that the correlation of profile peaks shows a curve with a slight fluctuation after an attenuation with interval *τ*, which represents the actual profile morphology as a random curve. The content of this section will lay the foundation for the numerical replacement of the actual morphology.

## 3. Numerical Simulation of Surface Morphology

Nowadays, the numerical simulation technique has become a major method to study tribology. Due to the fact that the workpiece surface is limited by the condition of processing, measuring and data collection, its use will be limited in the present analysis [33]. Meanwhile, with the deepening of research, the limitation of statistical characterizations of rough surfaces are more and more consequential; such characterizations cannot accurately express some local details and random features of surface morphologies. Therefore, the use of surface characteristic parameters to describe the profile is unable to express the real characteristics of the surface [34,35]. In order to more accurately and efficiently reflect the surface morphology, in this section, the fractal dimension *D* and scale coefficient *G* of the profile curve are obtained based on the measured surface profile data, and then an effective simulation of the surface profile is realized using fractal theory.

### 3.1. Fractal Simulation of Surface Profile

Up to now, studies on the structure of rough surfaces are mainly based on the Euclidean Geometry Model. However, many complicated surface structures cannot be clearly described by this model. According to the relevant literature [19], the surface profiles of machined parts have the characteristics of continuity, nondifferentiability and self-affinity in the mathematical sense. Mandelbrot constructs a fractal function to represent a rough surface profile according to the above characteristics, which is called the Weierstrass-Mandelbrot function [36]:

(9) $$z(x)=G^{\left(D-1\right)}\sum_{n=n_{l}}^{\infty }\gamma^{-(2-D)n}\cos(2\pi \gamma^{n}x)$$

where *z*(*x*) is the height of the random surface profile, *G* is the characteristic scale coefficient of the profile, *x* is the displacement coordinate of the profile and *D* is the fractal dimension, with the range being (1,2). Due to the randomness of surface profile, the minimum frequency determined by the sampling length *L*:$\gamma^{n}=1/L$; $n_{l}$ is an ordinal number, which depends on the cutoff frequency *ω* of the profile, representing the spatial frequency of the random profile.

**Figure 10:**
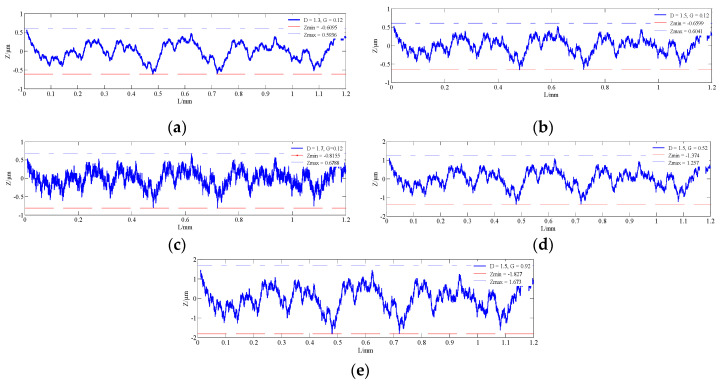
Two-dimensional rough surface curves with different *D* and *G* (**a**) D = 1.30, G = 0.12; (**b**) D = 1.50, G = 0.12; (**c**) D = 1.70, G = 0.12; (**d**) D = 1.50, G = 0.52. and (**e**) D = 1.50, G = 0.92.

According to Equation (9), two-dimensional morphology curves of machined rough surfaces are constructed using the M-B fractal function in order to show the effect of different fractal dimensions *D* (*D* = 1.3, 1.5, 1.7) on the curve when *G* = 0.12; at the same time, when the *D* = 1.5, the scale coefficient *G* is 0.12, 0.52 and 0.92 respectively, and when the sampling frequency *f* = 10,000, that is, 10,000 points are sampled per millimeter, *L* = 1.2 mm, and $n_{l}$ = 3. Two-dimensional rough surface curves with different *D* and *G* are shown in Figure 10.

As shown in Figure 10, when the scale coefficient *G* is constant, the fractal dimension *D* is related to the amplitude variation of the surface morphology. When the fractal dimension *D* is small, the microstructure is simple. As the fractal dimension *D* gradually becomes larger, the surface high-frequency component becomes more and more dense and the details are more and more abundant.

According to the simulation curve of a rough surface, the surface morphology is unsteady and stochastic. Therefore, this paper uses characterization theory to solve the morphological parameters, so as to observe the consistency between the theoretical morphology and the actual one. According to Equations (1)–(3), we can get the morphological parameters with different fractal dimensions *D* and different scale coefficients *G*. The specific values are listed in Table 2.

As can be seen in Table 2, when the scale coefficient *G* is constant, the maximum and minimum values of the profile curve, the arithmetic mean deviation *R_a_*, the profile height mean square error *R_q_* and the average variance *σ*^2^ show no distinct change with the increase of the fractal dimension *D*. However, when the fractal dimension *D* is constant, with *G* gradually becoming larger, the maximum and minimum value of the curve, *R_a_*, *R_q_* and *σ*^2^ show a significant increasing trend, which indicates that the scale coefficient *G* plays a decisive role in the amplitude of the surface morphology.

### 3.2. Characteristics Analysis of the Surface Profile

**Figure 11:**
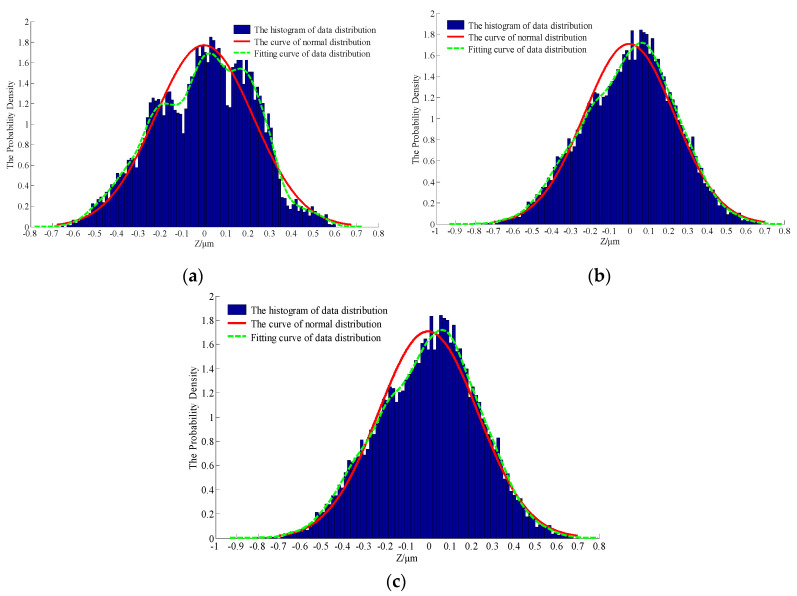
The probability density for different *D* and *G*: (**a**) *D* = 1.5, *G* = 0.12; (**b**) *D* = 1.7, *G* = 0.12; (**c**) *D* = 1.5, *G* = 0.52.

**Figure 12:**
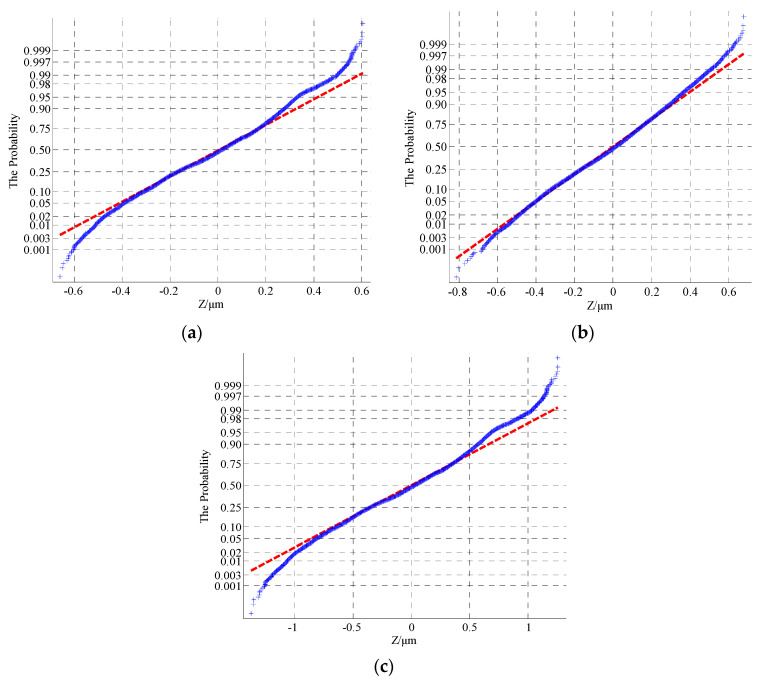
The probability density for different *D* and *G*: (**a**) *D* = 1.5, *G* = 0.12; (**b**) *D* = 1.7, *G* = 0.12; (**c**) *D* = 1.5, *G* = 0.52.

**Figure 13:**
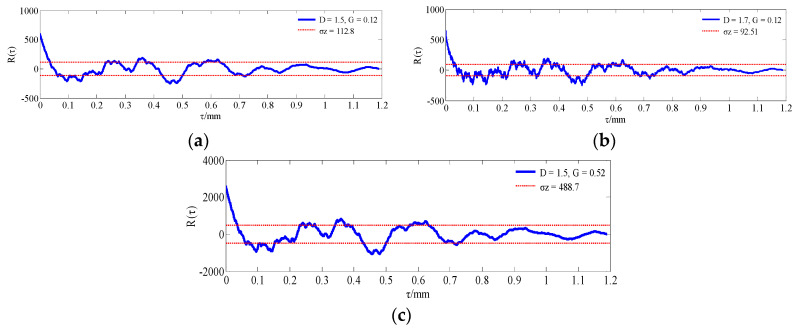
Autocorrelation graphs for different *D* and *G*: (**a**) *D* = 1.5, *G* = 0.12; (**b**) *D* = 1.7, *G* = 0.12; (**c**) *D* = 1.5, *G* = 0.52.

The morphological characterization parameters are only related to the relative height of the profile peak, so the surface geometry cannot be well described. In order to better reflect the characteristics of rough surfaces and increase the contrast between the simulated morphology and the actual profile, a statistical analysis of the probability density and autocorrelation function on the basis of the characteristic parameters was performed. According to the theoretical knowledge presented in Section 2, Figure 11, Figure 12 and Figure 13 show the probability density graphs, a QQ test figure and autocorrelation graphs for different *D* and *G*.

From Figure 11 and Figure 12, the surface profile data obtained by the fractal theory are basically in accordance with the normal distribution, which is basically consistent with the data distribution law obtained from the actual machining surface. In addition, it can be seen from Figure 13 that the correlation of profile peaks shows a curve with a slight fluctuation after an attenuation with the interval *τ*, which is also approximately consistent with the correlation law of the measured surface morphology. This section lays the foundation for the digital simulation and effective replacement of the current surface morphology characterization techniques.

## 4. Result Analysis

It is practical to realize a digital simulation of the actual morphology and replace the actual morphology with the simulated one. The digital substitution of the actual morphology not only provides an effective way for the establishment and analysis of the surface contact model, but also provides a new idea regarding the actual surface morphologies of heavily machined parts. In order to verify the validity of the simulated morphology instead of the actual morphology, this paper compares the morphological characterization parameters and the surface fractal dimension, and then realizes the mutual substitution between the simulated morphology and the actual one.

### 4.1. Comparison of Morphological Characterization Parameters

In this paper, grinding and milling specimens were selected as the experimental objects. Through the analysis of the morphological characterization parameters of the third and fourth sections, this section compares the arithmetic mean deviation *R_a_*, peak height mean square error *R_q_* and peak height average variance *σ*^2^ for the simulated morphologies (*D* = 1.5; *G* = 0.12, 0.22, 0.32, 0.92) and the actual machined surface (grinding and milling) of the same grade range. The specific comparison parameters are listed in Table 3.

As shown in Table 3, when the fractal dimension *D* = 1.5, the simulated morphology parameters are basically consistent with the characterization parameters of the measured morphology, i.e., the error percentage of arithmetic mean deviation *R_a_*, peak height mean square error *R_q_* and peak height average variance *σ*^2^ are smaller than 10%. Therefore, from the morphological characterization parameters, the actual surface morphology of grinding and milling can be replaced by a fractally simulated morphology.

### 4.2. Comparison of Fractal Dimension

According to [37], for the profile curve of fractal rough surface, there is a specific exponential relationship between the power spectrum *P*(*ω*) and the cutoff frequency *ω*:

(10) $$P\left(\omega \right)={\left|\omega \right|}^{-\left(5-2D\right)}$$

Then, the logarithm operation is performed on both sides of Equation (10), and the relationship curve between log *P*(*ω*)and log*ω* is fitted. If the logarithmic slope of the double logarithmic power spectrum is *K* = −(5 − 2*D*), the fractal dimension *D* can be obtained as:

(11) $$D=\frac{5+K}{2}$$

where 1 < *D* < 2, so, −3 < *K* < −1.

In practical applications, the power spectrum *P*(*ω*) is usually obtained by discrete Fourier transform based on the sampled data. Then, the logarithmic power spectra log *P*(*ω*) ~ log (*ω*) and its slope are obtained. Finally, the fractal dimension *D* is obtained according to Equation (11).

**Figure 14:**
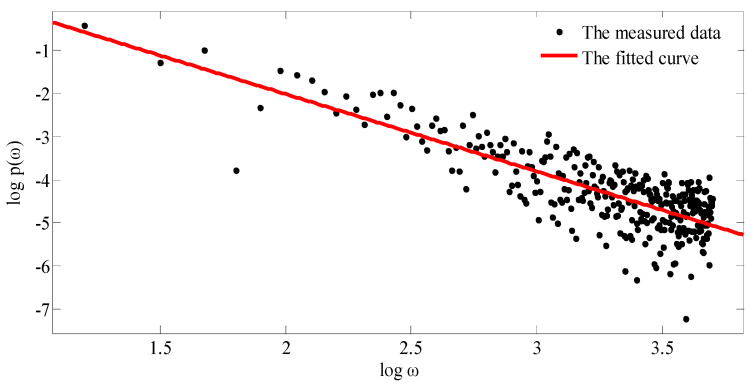
Double logarithmic power spectrum curve.

Table 4 shows the morphology power spectrum data of the measured grinding surface (*R_a_* = 0.2374); the double logarithmic power spectrum curve is shown in Figure 14, from which the curve slope *K* is obtained. According to the Equation (11), the fractal dimension *D* is:

(12) $$D=\frac{5+K}{2}=\frac{5-2.1086}{2}=1.4457$$

The fractal dimension *D* of the measured grinding surface (*R_a_* = 0.1815) and the measured milling surface (*R_a_* = 0.3067, 0.5365) can be obtained by the same method. The error percentages of fractal dimension *D* between the actual morphology and the simulated morphology are listed in Table 4.

The results show that the fractal dimension of the measured morphology is basically consistent with the simulated morphology using the same morphology characterization parameters, i.e., the error is less than 6%, which reflects a high consistency. Therefore, the actual surface morphology of the grinding and milling can be replaced by a simulated surface with a specific fractal dimension.

## 5. Conclusions

In this paper, a fractal surface profile simulation method based on measured data is presented. The research and methods provide a new way to analyze the contact performance between the morphology features and the interface, and to improve the morphology design and assembly quality. This article compares the characterization parameters, autocorrelation and fractal dimension of the simulated and actual morphologies, and reached the following conclusions:

1. The probability density statistics, the QQ test chart and the autocorrelation are used to analyze the profile data distribution law of the simulated surface and the actual surface, respectively. The results show that the profile data of the simulated surface and the actual machined surface basically conform to a normal distribution.
2. From the perspective of the morphology characterization parameters, when the fractal dimension *D* = 1.5, the morphology parameters of the simulated surface are basically consistent with those of the actual machined surface, and the error percentage of arithmetic mean deviation *R*a, the peak height mean square error *Rq* and the peak height average variance *σ*^2^ are all within 10%.
3. The calculated value of the measured fractal dimension is basically consistent with the given fractal dimension of the simulated morphology, i.e., the error is within 6%, which shows that the measured morphology and the simulated morphology have a high degree of consistency.

To sum up, for parts machined by grinding and milling, the surface morphology can be approximately replaced by a simulated morphology generated by fractal theory. In order to further expand the practicability and simulation scope of the proposed fractal simulation method, two aspects of work need to be carried out: (1) further expand the application scope of the 2D fractal simulation method to enhance its practicability; (2) In order to realize the effective simulation of 3D surface morphology of parts, related research on 3D fractal simulation methods will be carried out.

The research presented in this paper provides a new way to effectively explain the relationship between surface morphology characteristic parameters and the interface contact performance, thereby making it possible to improve the surface design and assembly quality of parts.

## Figures and Tables

**Table 1:** Characterization parameters of specimen surface morphology.

Specimen	*R_a_* (μm)	*R_q_* (μm)	*σ*^2^ (μm)
1	0.1825	0.2343	0.0539
2	0.2374	0.3093	0.0956
3	0.3067	0.3963	0.1570
4	0.5365	0.6650	0.4397

**Table 2:** The characterization parameters of surface profile with different *D* and *G.*

*D*	*G*	*R_a_* (μm)	*R_q_* (μm)	*σ*^2^ (μm)
1.30	0.12	0.1953	0.2371	0.0562
1.50	0.12	0.1832	0.2246	0.0505
1.70	0.12	0.1873	0.2328	0.0542
1.50	0.52	0.3813	0.4676	0.2187
1.50	0.92	0.5072	0.6220	0.3869

**Table 3:** Comparison parameters of simulated and practical morphologies.

Morphology Parameter	Simulated Morphology (*D* = 1.5)	Measured Morphology		Error Percentage (%)
*R_a_* (μm)	0.1892	0.1815	Grinding	4.24
	0.2480	0.2374		4.47
	0.2991	0.3067	Milling	2.48
	0.5072	0.5365		5.46
*R_q_* (μm)	0.2246	0.2343	Grinding	4.14
	0.3042	0.3099		1.84
	0.3668	0.3963	Milling	7.44
	0.6220	0.6650		6.47
*σ*^2^ (μm)	0.0505	0.0539	Grinding	6.31
	0.0925	0.0956		3.24
	0.1416	0.1503	Milling	5.79
	0.3969	0.4326		8.25

**Table 4:** Error percentages of simulated and actual morphologies.

Measured Morphology *R_a_* (μm)	Measured Morphology (*D*)	Simulated Morphology (*D*)	The Error Percentage (%)
0.1815	1.42	1.50	5.33
0.2374	1.44	1.50	4.00
0.3067	1.48	1.50	1.33
0.5365	1.45	1.50	3.33
